# Morphological and Taxonomic Properties of *Tokyovirus*, the First *Marseilleviridae* Member Isolated from Japan

**DOI:** 10.1264/jsme2.ME16107

**Published:** 2016-11-19

**Authors:** Masaharu Takemura

**Affiliations:** 1 Laboratory of Biology, Department of Liberal Arts, Faculty of Science, Tokyo University of Science (RIKADAI)Shinjuku-ku, Tokyo 162–8601Japan

**Keywords:** giant virus, *Marseilleviridae*, virus isolation, phylogenetic analysis, genome analysis

## Abstract

Members of the *Marseilleviridae* family are large DNA viruses with icosahedral particle structures that infect *Acanthamoeba* cells. The first *Marseillevirus* to be discovered was isolated in 2009. Since then, several other members of the *Marseilleviridae* family have been reported, including *Lausannevirus*, *Senegalvirus*, *Cannes 8 virus*, *Insectomime virus*, *Tunisvirus*, *Melbournevirus*, *Port-Miou virus*, and *Brazilian Marseillevirus*, which have been isolated from Europe, Africa, Australia, and South America. The morphological and genomic properties of a new *Marseilleviridae* family member, *Tokyovirus*, discovered in a water/soil sample from a Japanese river in Tokyo, were described in the present study. *Tokyovirus* possesses icosahedral particles of up to 200 nm in diameter, as revealed by a transmission electron microscopy (TEM) analysis, which form a giant virion factory in *Acanthamoeba* cells. A preliminary genome analysis predicted 487 coding sequences. A dot plot analysis and phylogenetic analysis using family B DNA polymerase, proliferating cell nuclear antigen (PCNA), and DNA-directed RNA polymerase alpha subunit genes revealed that *Tokyovirus* shares similarities with *Marseillevirus*, *Melbournevirus*, and *Cannes 8 virus* (*Marseilleviridae* subclade A), but not with *Lausannevirus* and *Port-Miou virus* (subclade B), *Tunisvirus* and *Insectomime virus* (subclade C), or *Brazilian Marseillevirus* (subclade D), suggesting that *Tokyovirus* has evolved separately from the previously described *Marseilleviridae* members.

So-called “giant viruses” are generally defined as double-stranded DNA viruses with particle diameters of larger than 200–300 nm, allowing them to be viewed under a light microscope, and a genome longer than 300 kbp (1). The discovery of *Acanthamoeba polyphaga mimivirus* (APMV), infecting *Acanthamoeba* cells, in a cooling tower in Bradford (UK) in 2003 revealed the existence of these giant viruses in commonly encountered environments (15, 22). These APMV were found to possess icosahedral particles of 750 nm in diameter and encode DNA genomes of 1.2 Mbp, both of which were significantly larger in size than all previously discovered viruses (15, 22). The number of APMV continue to expand because studies on these “giant viruses” have revealed the existence of *A. castellanii mamavirus*, *A. polyphaga moumouvirus*, *Cafeteria roenbergensis virus*, and *Megavirus chilensis*, all of which belong to the family *Mimiviridae* (1, 6, 9, 14, 28). Additionally, studies on “giant viruses” have facilitated the identification of giant viruses of three other types. The first is a group of giant viruses with amphora-shaped particles of approximately 1 μm in diameter, which includes the *Pandoraviruses* (*Pandoravirus salinus*, *P. dulcis*, and *P. innopinatum*), *Pithovirus sibericum*, and *Mollivirus sibericum* (17, 18, 20). The second type is a recently discovered giant virus lineage, *Faustovirus*, closely related to *Asfarviridae* (23). The third is a group of giant viruses with particles smaller than the other *Mimiviridae*, diameters of 200 nm, and genomes of 300 kbp to 400 kbp, and has been named the family *Marseilleviridae* (10). To date, several DNA viruses of the *Marseilleviridae*, which were discovered as *Marseillevirus* in a cooling tower in Paris (8), have been reported to reside in a number of locations including rivers, the human gut, and insect bodies. They include *Lausannevirus* from the Seine River (France), *Senegalvirus* from an African human gut microbiota, *Cannes 8 virus* from a cooling tower in Cannes (France), *Tunisvirus* from fountain water in Tunis (Tunisia), *Insectomime virus* from insect larvae (*Eristalis tenax*) in Tunisia, *Melbournevirus* from a freshwater pond in Melbourne (Australia), *Port-Miou virus* from a submarine spring in the Cassis Port-Miou Calanque, and *Brazilian Marseillevirus* from a sewage sample from Brazil (2, 3, 7, 11–13, 16, 27). *Marseillevirus*-like viruses have also been found in healthy human blood, suggesting that giant viruses are part of the human blood virome (21). However, another research group in the US reported that they were unable to detect *Marseillevirus*-like viruses in plasma from healthy human blood (19).

This study describes the discovery of a new virus of the family *Marseilleviridae*, isolated from a water/soil sample from the Arakawa River, located in the eastern area of Tokyo, Japan, which is the first giant virus isolated in Japan. It has been named *Tokyovirus*, according to the conventional manner to name *Marseilleviridae*, namely, the city in which they were firstly isolated or analyzed forms their name. Morphological and genome analyses of *Tokyovirus* and comparisons with other members of the *Marseilleviridae* reported to date were also performed.

## Materials and Methods

### Culture of amoeba cells

*A. castellanii* (Douglas) Neff (ATCC 30010™) cells were purchased from the American Type Culture Collection (ATCC, Manassas, VA, USA) and cultured in PYG medium (Proteose peptone, 2% [w/v]; yeast extract, 0.1% [w/v]; 0.4 mM CaCl_2_; 4 mM MgSO_4_·7H_2_O; 2.5 mM Na_2_HPO_4_·7H_2_O; 2.5 mM KH_2_PO_4_; sodium citrate·2H_2_O, 0.1% [w/v]; 0.05 mM Fe(NH_4_)_2_(SO_4_)_2_·6H_2_O; 100 mM glucose; pH 6.5) at 26°C according to the ATCC protocol. In order to avoid contamination of the culture by bacteria and fungi, three antibiotics were added to the culture: 100 μg mL^−1^ of penicillin–streptomycin (GIBCO/Thermo Fischer Scientific, Yokohama, Japan), 100 μg mL^−1^ of ampicillin (Wako Chemicals USA, Richmond, VA, USA), and 5 μg mL^−1^ of amphotericin B (GIBCO) (20).

### Virus isolation

A water/soil sample was collected from the Arakawa River, located in the eastern area of Tokyo, Japan (35°41′54.21″N, 139°51′18.41″E). After the removal of mud by filtration through filter paper with a pore size of 20 μm (43; Whatman International, Maidstone, UK), the sample was further filtered through filter paper with a pore size of 0.8 μm (Millex-AA; Merck Millipore, Darmstadt, Germany). The filtered sample was concentrated by polyethylene glycol (PEG) precipitation overnight at 4°C (final concentration: PEG 10,000, 8% [w/v]; NaCl, 0.48% [w/v]), followed by centrifugation at 1,500×*g* at 4°C for 30 min (20). After removal of the supernatant, the pellet (invisible) was resuspended in 4 mL of PYG, and filtered again through filter paper with a pore size of 0.8 μm (Millex-AA; Merck Millipore). Four milliliters of fresh PYG and 1 mL of an amoeba cell suspension were added to this viral solution, and the solution was divided and cultured into 56 wells on a 96-well culture plate at 26°C. After 10 d, amoeba cells were detected in only one of the 56 wells. These cells showed delayed proliferation, and were almost round in shape. The culture supernatant from this one well was inoculated into fresh amoeba cells in three wells of a 96-well culture plate. After 1 week, almost all cells appeared to be round in shape. The supernatant was then inoculated into fresh amoeba cells in one well of a 12-well culture plate. After 4 d, almost all cells appeared to be round in shape. The supernatant was inoculated into fresh amoeba cells in a 25-cm^2^ culture flask. After 2 d, rounded amoeba cells were harvested for observations by transmission electron microscopy. The supernatant was stored at 4°C as an isolated virus solution.

### Electron microscopic observation

Harvested cells infected by *Tokyovirus* were washed twice with PBS, fixed with 2% glutaraldehyde solution at 4°C overnight, and then transferred to fresh 2% glutaraldehyde solution. Fixed cells were washed three times with PBS and then stained with 2% osmium tetroxide at 4°C for 1 h. Osmium-stained cells were dehydrated in increasing ethanol concentrations (50%, 70%, 80%, 90%, 95%, and 100%), each at room temperature for 5 min, and embedded in Epon-812 (TAAB Laboratory Equipment, Berks, UK). The hardening of Epon-812 took 2 d at 60°C. Ultra-thin sections of 80 nm were obtained using a microtome (Leica Microsystems, Tokyo, Japan), and were stained with 2% uranyl acetate for 10 min, followed by lead citrate for 5 min. Observations were performed using TEM (JEM-1400; JEOL, Tokyo, Japan, or H-7600; Hitachi, Tokyo, Japan).

### Visualization of virion factories (VF)

Cultured amoeba cells on a coverslip in a 12-well microplate infected by *Tokyovirus* at 8 h post-infection (p.i.) were washed twice with PBS, and fixed with methanol at room temperature for 10 min. Fixed cells on a coverslip were washed twice with PBS, and then completely air-dried. Dried cells were incubated with 500 ng mL^−1^ of DAPI for 50 s and then immediately washed twice with PBS. Stained cells on the coverslip were mounted in Vectashield (Vector Laboratories, Burlingame, CA, USA) and visualized using a fluorescence microscope (BX50; Olympus, Tokyo, Japan).

### Virus cloning

Virus cloning was performed according to a cloning method used for *Mollivirus* (18), with several modifications as described below. Amoeba cells were seeded into three wells in a 12-well culture plate with 1 mL of PYG. A total of 0.5 mL of the isolated virus solution was added to each well, and 1 h after the inoculation, excess viruses were removed. The cells were washed three times with 1 mL of PYG, then harvested by scraping. Three serial dilutions were performed in the next nine wells by mixing 100 μL of the previous well with 100 μL of fresh PYG. The last three dilutions in each case were observed by light microscopy to verify that there were fewer than two amoeba cells in each well. Observations revealed that there were 9, 8, and 2 amoeba cells in each well. Hundreds of fresh amoeba cells were added to the well containing only two cells and were cultured for 3 d until almost all cells became rounded. The viral clone obtained was then amplified and stored for later use, and genomic DNA extraction was performed as described below.

### Genome analysis

After virus cloning, the genomic DNA of *Tokyovirus* (1.1 μg) was prepared from PYG culture media containing viral particles according to the manufacturer’s protocol (NucleoSpin^®^ Tissue; Macherey-Nagel GmbH and Co. KG). A DNA library for sequencing was prepared and sequencing was performed as described previously (25). A DNA library for sequencing was prepared using a TruSeq Nano DNA LT library prep kit (Illumina, San Diego, CA, USA), and sequencing was performed on a HiSeq 2500 platform (Illumina). The total number of reads was 49,062,650 (each of the reads had a length of 100 nucleotides). Edena software was used for the assembly of 1,000,000 reads into 68 contigs. Contigs had an average length of 5,481 nucleotides, and the maximum contig had a length of 360,777 nucleotides. The total length of the 68 contigs was 372,707 nucleotides (25). The mapping of reads was performed using the software GeneData Expressionist for Genomic Profiling version 9.1.4a and BWA-MEM, according to the manufacturer’s protocol, and was visualized and confirmed using the software Integrative Genomic Viewer (Broad Institute, Cambridge, MA, USA). The prediction of gene function was conducted using NCBI blastp in the NCBI nr and NCBI COG databases. A prediction of the coding region of the *Tokyovirus* genome was conducted using CRITERIA version 1.05b and Glimmer 2 version 2.10. A prediction of tRNA was conducted using tRNAScan-SE version 1.23, according to the manufacturer’s protocol (25). A genome analysis including dot plots for genome comparisons and a Venn diagram to compare gene contents were performed using IMC genomics software (In Silico Biology, Yokohama, Japan).

### Phylogenetic analysis

The amino acid sequences of the B-family DNA polymerase of eight *Marseilleviridae* family members and APMV were obtained from NCBI protein sequence databases (http://www.ncbi.nlm.nih.gov/). Accession numbers for all sequence data used for this study are shown in Table S1. Sequences were aligned using the ClustalW program implemented in the MEGA7 software (ver. 7.0.14) (26) with default parameters. A maximum-likelihood inference program and the LG model was used as a substitution model with discrete gamma-distributed rate variations and a proportion of sites being invariant (24) to construct the phylogenetic tree. In order to estimate branch support, 100 bootstrap replications were performed. In another analysis, a phylogenetic tree was reconstructed using 30 B-family DNA polymerases, for example from *Mimiviridae*, *Poxviridae*, and *Pandoraviruses*, as described above (Table S1). Additionally, the amino acid sequences of the proliferating cell nuclear antigen (PCNA) and DNA-directed RNA polymerase alpha subunit of nine *Marseilleviridae* family members and APMV were obtained from NCBI protein sequence databases (Table S2 and S3), and a phylogenetic tree was constructed as described above.

### Sequence data

The almost complete genomic sequence of *Tokyovirus* has been deposited in DDBJ/ENA/GenBank under accession number AP017398 (25).

## Results and Discussion

### Morphological features of *Tokyovirus*

Giant viruses in the narrow sense contain several groups: *Mimiviridae*, *Marseilleviridae*, *Faustovirus*, and the amphora-like giant viruses *Pandoravirus*, *Pithovirus*, and *Mollivirus* (4). The particle sizes of these giant viruses are widely diversified, from *Marseilleviridae* (approximately 200 nm in diameter) to *Pithovirus* (1.5 μm in diameter). *Tokyovirus* particles were isolated from a muddy freshwater sample from Arakawa River, located in east Tokyo. Arakawa River is one branch of the Tonegawa River, which runs through the largest valley in Japan. The amplification of *Tokyovirus* and the rounding of all amoeba cells took 2–3 weeks after the inoculation of amoeba cells. This result indicated that *Tokyovirus* particles were not numerous in the water/soil samples tested. However, it is possible that *Tokyovirus* is widely distributed throughout Arakawa River and Tonegawa valley. Moreover, other unknown *Marseilleviridae* family members may inhabit widely separated rivers in Japan.

**Fig. 1:**
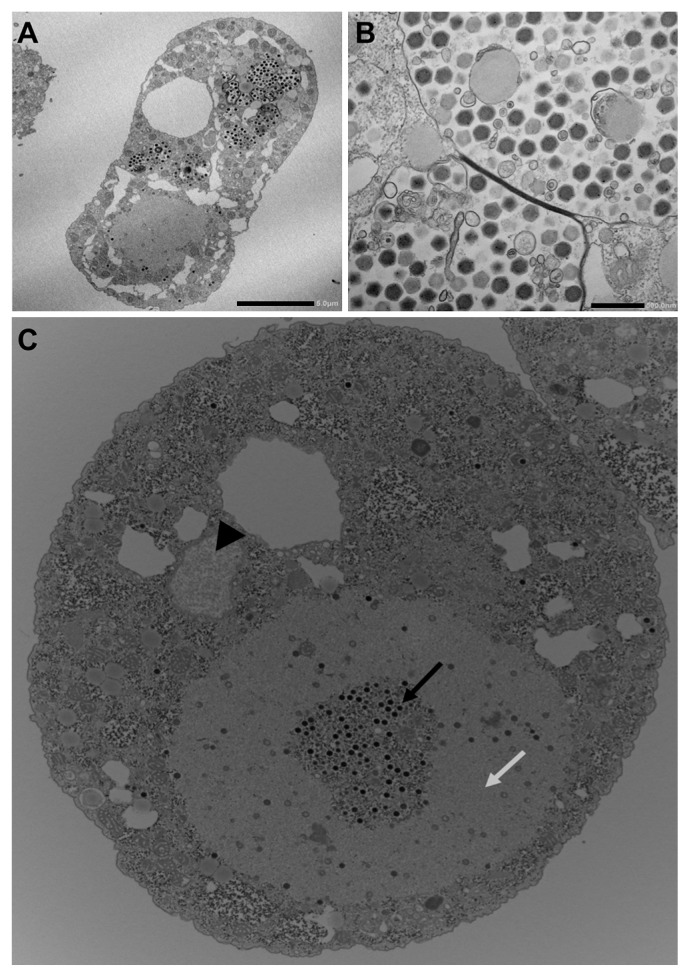
Transmission electron microscopy images of ultrathin sections of *Tokyovirus*-infected *Acanthamoeba castellanii* cells. (A) Comprehensive image of a *Tokyovirus*-infected amoeba cell at 8 h p.i. Mature virions (small black particles) are encapsulated in several intracytoplasmic vacuoles. A putative virion factory is seen in the lower-left portion of the cell. The cell nucleus is not evident in this section. Scale bar, 5 μm. (B) Enlarged image of *Tokyoviruses* in the intracytoplasmic vacuoles of the amoeba. The particle diameter is approximately 200 nm. Scale bar, 500 nm. (C) Transmission electron microscopy image of a typical virion factory in the cytoplasmic region of *Tokyovirus*-infected amoeba cells. Virion factories are distinctly classified into two portions: one in which mature virions densely co-exist (black arrow), and another portion surrounding the former (white arrow). In this section, a small part of the cell nucleus is shown (black arrowhead).

Rounded cells were subjected to an electron microscopic analysis, which revealed that many *Marseillevirus*-like particles were present in amoeba cytoplasmic vacuoles. Observations by TEM of infected amoeba cells at 8 h p.i. revealed intracellular icosahedral particles of approximately 200 nm in diameter, similar to those of *Marseilleviridae* such as *Marseillevirus* and *Melbournevirus*, with no surrounding fibrils, similar to APMV and *Megavirus* particles. *Tokyovirus* particles accumulated in intracytoplasmic vacuoles as with other *Marseilleviridae*, including putative mature particles (Fig. 1A and B). The main cytopathic effects caused by *Tokyovirus* against amoeba cells were the rounding of cells and suspension in culture media. The prominent destruction of cells, such as that caused by APMV, was not observed. *Tokyovirus* infection was accompanied by the large-scale development of VF in the amoeba cytoplasm, similar to that observed with other *Marseilleviridae* members (8, 10). A recent study reported that *Marseillevirus* possesses unique mechanisms for entry into *Acanthamoeba* cells, forming giant infectious vesicles surrounded by membranes, which differs from the entry mechanisms of other giant viruses (5). *Tokyovirus* was also found to display several morphological types in *Acanthamoeba* cells, such as giant vesicles including many viral particles surrounded by membranes and single particles in the amoeba cytoplasm (Fig. S1). These results suggest that this unique entry mechanism into host cells is a universal characteristic of *Marseilleviridae*.

**Fig. 2:**
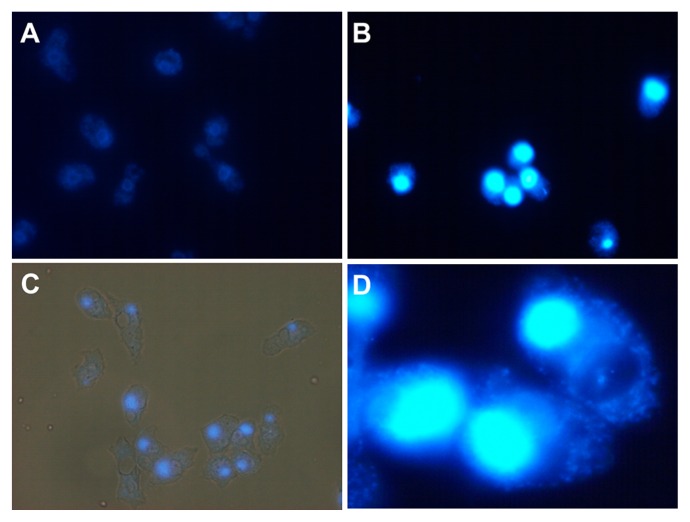
Fluorescent microscopy images of *Tokyovirus*-infected *Acanthamoeba castellanii* cells stained with DAPI. Staining by DAPI was performed at 8 h p.i., as described in the Materials and Methods. (A) Control amoeba cells without *Tokyovirus* infection. Cell nuclei were slightly stained and appeared donut-shaped. (B) *Tokyovirus*-infected amoeba cells. Virion factories were brightly stained. (C) Merged view of fluorescent and phase-contrast images. (D) Enlarged image of *Tokyovirus*-infected amoeba cells for which the virion factory is brightly stained.

Observations by TEM of the VF of *Tokyovirus* at 8 h p.i. revealed its detailed morphological features. As for *Marseillevirus*, no remarkable membranous structures were observed at the periphery of the VF (Fig. 1C). The typical morphology of the VF consisted of two clearly divided areas: one in which mature viral particles were abundantly present and the other in which fewer viral particles were present proceeding construction. In the latter area, capsid assembly and DNA encapsidation both proceeded simultaneously (Fig. S2), as observed for *Marseillevirus* (8). DAPI staining of a VF from an infected amoeba cell at 8 h p.i. revealed that it was larger than the amoeba cell nucleus, in many cases occupying 1/3 of the amoeba cytoplasm (Fig. 1C, 2, S3, Table S4 and S5), as observed for other *Marseilleviridae* members (5, 8).

### Genome analysis

*Tokyovirus* has a 360–370 kb genome, of which more than 372,707 bp corresponds to the total contig length (25). A genome analysis using the Illumina HiSeq system showed that the maximum contig was 360,777 nucleotides in length as described above, and was located from 8,609 to 369,355 in deposited genome data under accession number AP017398. There are 43 contigs located from 1 to 8,608, and 24 contigs located from 369,356 to 372,707; therefore, the sequences of these terminal regions have not yet been solved. Nevertheless, this size of the total contig length (372,707) is roughly equivalent to the genome size of other *Marseilleviridae* family members, such as *Marseillevirus* (368,454), *Cannes 8 virus* (374,041), *Melbournevirus* (369,360), and *Lausannevirus* (346,754) (2, 8, 12, 27). This nearly complete genome sequence was predicted to contain 487 coding sequences (CDSs) including genes for the 3Rs (replication, recombination, and repair), transcription, amino acid transport and metabolism, histones, and two tRNA genes (one of which is a pseudogene) (25). For example, histone H2A (fused to H2B) and H3-like genes were respectively found on the *Tokyovirus* genome, similar to other *Marseilleviridae*. Several translation-related genes such as translation initiation factor and translation elongation factor genes were also found on the *Tokyovirus* genome, similar to *Cannes 8 virus* and *Melbournevirus*. One CDS was more similar to an unknown CDS of *M. sibericum* than that of other *Marseilleviridae*, and another was similar with an unknown CDS of *P. dulcis*, similar to other *Marseilleviridae*. Most CDSs exhibited high homology with other *Marseilleviridae* family members. On the other hand, several putative CDSs were found to be *Tokyovirus*-specific CDSs, namely, they did not exist on other *Marseilleviridae* genomes, which was revealed by a blastp search. The functions of these putative CDSs specific for *Tokyovirus* have not yet been elucidated.

**Fig. 3:**
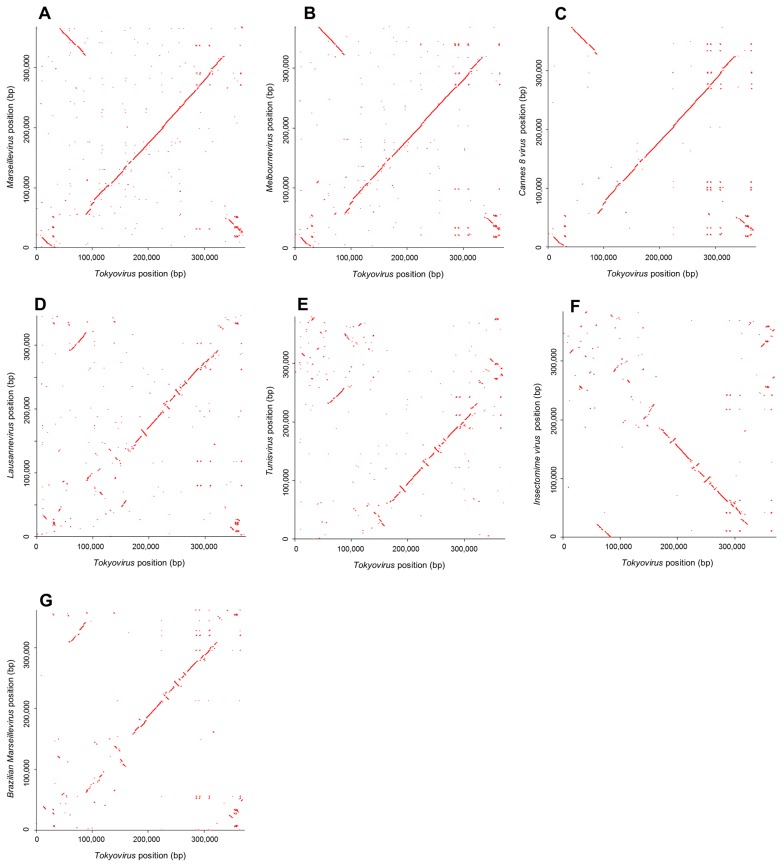
Genome comparison of *Tokyovirus* and seven other *Marseilleviridae* family members using a dot plot analysis. (A) Dot plots based on the genomic positions of orthologous CDSs between *Tokyovirus* and *Marseillevirus*. The first part of the *Tokyovirus* genome exhibits collinearity with the final part of the *Marseillevirus* genome, but in the opposite orientation. The large central parts of both genomes exhibit clear collinearity. (B) A dot plot analysis between *Tokyovirus* and *Melbournevirus*. Almost the same collinearity was shown as that in A. (C) A dot plot analysis between *Tokyovirus* and *Cannes 8 virus*. Almost the same collinearity was shown as those in A and B. (D) A dot plot analysis between *Tokyovirus* and *Lausannevirus*. The former part of the *Tokyovirus* genome (position approximately 170,000 bps) exhibits weak collinearity with the *Lausannevirus* genome, whereas the latter part (position approximately 170,000–320,000 bps) shows some collinearity, with several inverted segments. (E) A dot plot analysis between *Tokyovirus* and *Tunisvirus*. The former part of the *Tokyovirus* genome exhibits weak collinearity, as with *Lausannevirus*. The latter part shows some collinearity with several inverted segments, similar to *Lausannevirus*. (F) A dot plot analysis between *Tokyovirus* and *Insectomime virus*. The former part of the *Tokyovirus* genome exhibits weak collinearity with the *Insectomime virus* genome, and the latter part shows some collinearity, but in the opposite orientation. (G) A dot plot analysis between *Tokyovirus* and *Brazilian Marseillevirus*. The former part of the *Tokyovirus* genome exhibits weak collinearity, as with *Lausannevirus* and *Tunisvirus*. The latter part shows some collinearity with several inverted segments, similar to *Lausannevirus* and *Tunisvirus*.

Dot plots to compare Tokyovirus with seven other Marseilleviridae family members indicated that the Tokyovirus genome was more similar to Marseillevirus, Melbournevirus, and Cannes 8 virus than to Lausannevirus, Tunisvirus, Insectomime virus, and Brazilian Marseillevirus (Fig. 3). The Marseilleviridae family has been divided into four subclades: A, B, C, and D (2, 10, 11, 13). Subclade A includes Marseillevirus, Cannes 8 virus, Senegalvirus, and Melbournevirus. Subclade B includes Lausannevirus and Port-Miou virus. Subclade C includes Tunisvirus and Insectomime virus. Subclade D includes the newly-found Brazilian Marseillevirus. A dot plot analysis suggested that Tokyovirus was similar to subclade A viruses, such as Marseillevirus, Melbournevirus, and Cannes 8 virus because the central part of the Tokyovirus genome showed greater collinearity with these subclade A Marseilleviridae genomes (Fig. 3A, B, and C) than with subclade B Lausannevirus (Fig. 3D), subclade C Tunisvirus and Insectomime virus (Fig. 3E and F), and subclade D Brazilian Marseillevirus (Fig. 3G). These genomic differences revealed by dot plot analyses between Tokyovirus and other Marseilleviridae are similar to those between previously reported Marseilleviridae of one subclade and that of other subclades (Fig. S4). On the other hand, between Marseilleviridae viruses, both of which were classified into the same subclade, a dot plot analysis hardly detected their differences (Fig. S5), as described previously (12), except for the case of subclade C (Fig. S5E).

It is of particular interest that a part of the *Tokyovirus* genome is inverted relative to that in subclade A *Marseilleviridae* genomes (Fig. 3A, B, and C). The region spanning approximately 40,000–90,000 bp of the *Tokyovirus* genome corresponded to the inverted region positioned around 320,000–370,000 of subclade A *Marseilleviridae* genomes. These results suggest that the genomic composition of *Tokyovirus* has evolved differently from *Marseillevirus*, *Melbournevirus*, and *Cannes 8 virus*. Although only 1 isolate of *Tokyovirus* has been obtained to date, *Tokyovirus* needs to be classified into a new subclade “E”, similar to the recently isolated *Brazilian Marseillevirus*, which is solely classified into subclade D (11).

**Fig. 4:**
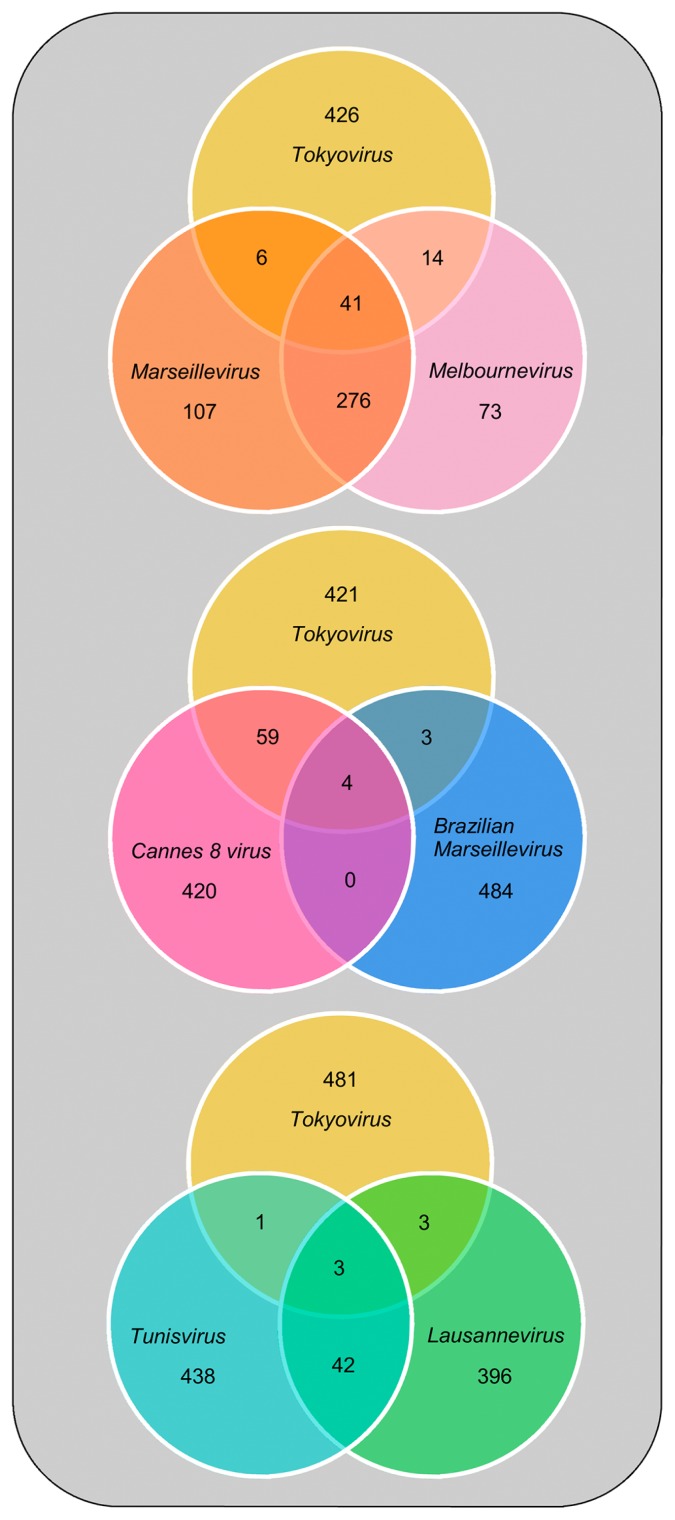
Venn diagram showing a comparative analysis of gene contents of *Tokyovirus* with six other *Marseilleviridae* family members. The numbers of genes showing >90% similarity are shown in the merged regions. The upper diagram shows an analysis of *Tokyovirus*, *Marseillevirus*, and *Melbournevirus*. The middle diagram shows an analysis of *Tokyovirus*, *Cannes 8 virus*, and *Brazilian Marseillevirus*. The lower diagram shows an analysis of *Tokyovirus*, *Tunisvirus*, and *Lausannevirus*.

Venn diagrams showed that *Tokyovirus* possessed more CDSs with >90% sequence identity to those of *Cannes 8 virus* (63 CDSs), *Marseillevirus* (47 CDSs), and *Melbournevirus* (55 CDSs), than to *Brazilian Marseillevirus* (7 CDSs), *Lausannevirus* (6 CDSs), and *Tunisvirus* (4 CDSs) (Fig. 4). The number of CDSs of *Tokyovirus* with >90% sequence identity to *Marseillevirus* and *Melbournevirus* was less than that of *Marseillevirus* and *Melbournevirus* (Fig. 4). This result supports the suggested classification of *Tokyovirus* into a new subclade (Fig. 4).

### Phylogenetic analysis

**Fig. 5:**
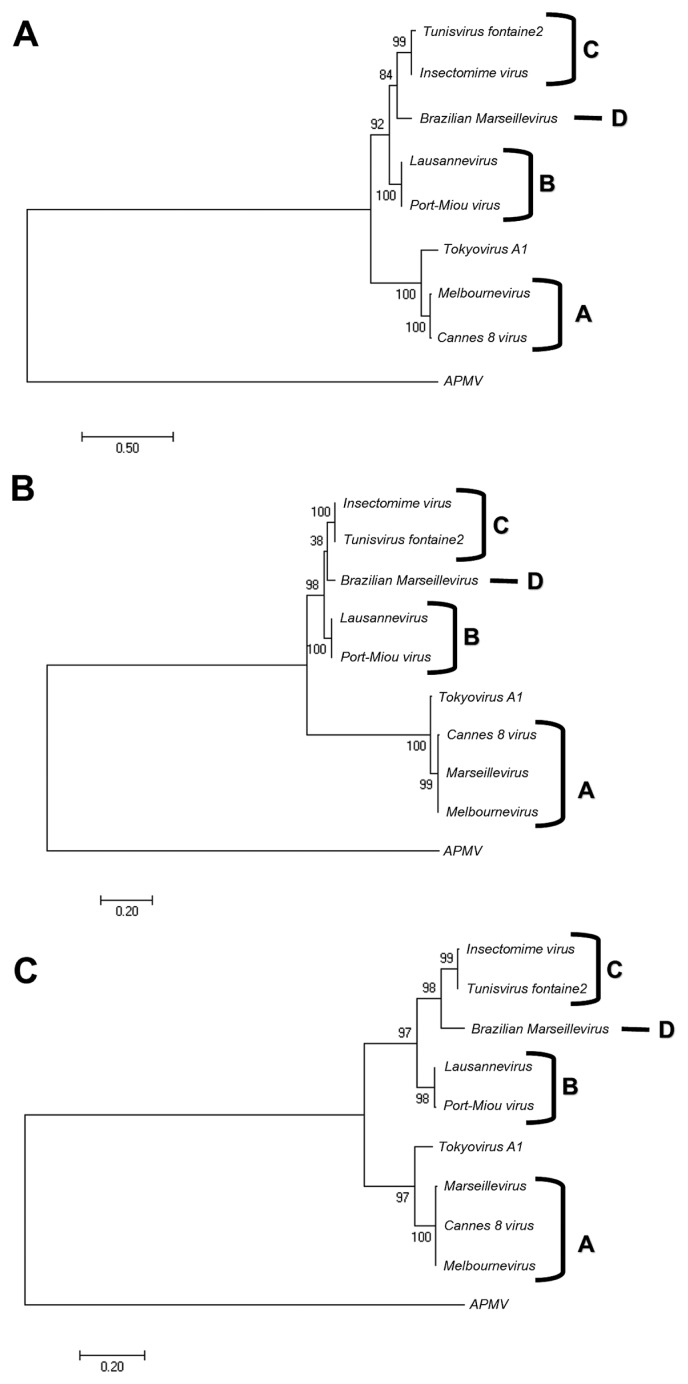
Unrooted maximum-likelihood phylogenetic trees of B-family DNA polymerase (A), PCNA (B), and DNA-directed RNA polymerase alpha subunit (C) sequences constructed using MEGA7 software (26). The trees were reconstructed based on alignments (DNA polymerase, 1,103 sites; PCNA, 257 sites; RNA polymerase alpha subunit, 452 sites) derived from the full-length alignment in which any column containing a gap was discarded. Numbers at the branch points denote percent bootstrap values. The accession numbers of respective sequences are listed in Tables S1~S3. The letters A, B, C, and D indicate the subclades of *Marseilleviridae*.

In all of the unrooted phylogenetic trees based on the genes of B-family DNA polymerase, PCNA, and DNA-directed RNA polymerase subunit alpha, *Marseilleviridae* were grouped into five subclades. Four of the five subclades corresponded to subclade A (*Marseillevirus*, *Melbournevirus*, and *Cannes 8 virus*), subclade B (*Lausannevirus* and *Port-Miou virus*), subclade C (*Tunisvirus* and *Insectomime virus*), and subclade D (*Brazilian Marseillevirus*) (Fig. 5 and S6). A phylogenetic analysis based on the B-family DNA polymerase gene revealed that *Tokyovirus* is more closely related to *Melbournevirus* and *Cannes 8 virus*, both of which belong to subclade A, than to *Lausannevirus*, *Tunisvirus*, and *Insectomime virus* (Fig. 5A and S6). Other phylogenetic analyses using PCNA and DNA-directed RNA polymerase subunit alpha genes also confirmed the putative classification of *Tokyovirus* described above (Fig. 5B and C). These results indicate that *Tokyovirus* needs to be classified into a new subclade of *Marseilleviridae*, which is closer to subclade A than to the other subclades.

### Conclusion

Mature *Tokyoviruses* in *Acanthamoeba* cells have icosahedral particles of approximately 200 nm in diameter and a genome size of 370–380 kb, as reported previously for other *Marseilleviridae* family members. The VF of *Tokyovirus* become enlarged, in many cases occupying 1/3 of the amoeba cytoplasm. In these VF, densely stained regions are evident, within which abundant mature particles are visible. These characteristics are typical of *Marseilleviridae* family members. According to the genome analysis, *Tokyovirus* is closely related to subclade A of *Marseilleviridae*; however, its classification into a new subclade “E” is suggested. The mechanisms by which *Tokyovirus* evolved away from other *Marseilleviridae* family members have not yet been elucidated. Ecological studies on *Marseilleviridae*, isolated from various environments, are of important significance for clarifying the relationship between giant viruses and living organisms, including humans. The results of the present study may stimulate further studies into the worldwide distribution of these viruses and their functional significance.

## Supplementary Information
