# Supplementary material for: Morphological and Taxonomic Properties of Tokyovirus, the First Marseilleviridae Member Isolated from Japan
Source: Microbes Environ. 2016 Nov 19;31(4):442–8. doi: 10.1264/jsme2.ME16107 (PMC5158117; doi:10.1264/jsme2.ME16107)
Supplement: Supplementary file 1 [file 31_442_s1.pdf]

Table S1 Accession numbers of the 32 B-family DNA polymerase sequences used for this study in Figs. 5A and S6

Accession No.	Sequence name
AFM52356	*Megavirus terra1*
AFM52364	*Megavirus courdo5*
AEX61758	*Megavirus courdo7*
AEX62677	*Moumouvirus monve*
YP_003986825	*Acanthamoeba polyphaga mimivirus* (APMV)
AEQ60511	*Acanthamoeba castellanii mamavirus*
AHA45542	*Hirudovirus strain Sangsue*
ALR83902	*Niemeyer virus*
YP_003970130	*Cafeteria roenbergensis virus BV-PW1*
NP_048107	*Choristoneura biennis entomopoxvirus*
NP_039057	*Fowlpox virus*
YP_004821396	*Yoka poxvirus*
NP_042094	*Variola virus*
AAB59829	*Vaccinia virus*
ADX22900	*Monkeypox virus*
YP_008052566	*Phaeocystis globosa virus*
AHC54969	*Tunisvirus fontaine 2*
AHA45970	*Insectomime virus*
YP_009238893	*Brazilian marseillevirus*
YP_004347308	*Lausannevirus*
ALH07009	*Port-Miou virus*
YP_009255117	*Tokyovirus* (*Tokyovirus A1*)
YP_009094792	*Melbournevirus*
AGV01694	*Cannes 8 virus*
YP_009000951	*Pithovirus sibericum*
YP_002154715	*Feldmannia species virus*
AGE58955	*Paramecium bursaria Chlorella virus OR0704.2.2*
AGE57130	*Acanthocystis turfacea Chlorella virus NE-JV-3*
ALD62120	*Mollivirus sibericum*
YP_008436920	*Pandoravirus salinus*
AGO82327	*Pandoravirus dulcis*
YP_009120445	*Pandoravirus innopinatum*

Table S2 Accession numbers of the 10 PCNA sequences used for this study in Fig. 5B

Accession No.	Sequence name
AHA46099	*Insectomime virus*
AHC54833	*Tunisvirus fontaine 2*
YP_009238758	*Brazilian marseillevirus*
YP_004347173	*Lausannevirus*
ALH06885	*Port-Miou virus*
YP_009254994	*Tokyovirus A1*
AGV01569	*Cannes 8 virus*
YP_003406953	*Marseillevirus*
AIT54803	*Melbournevirus*
AAQ09580	APMV

Table S3 Accession numbers of the 10 DNA-directed RNA polymerase sequences used for this study in Fig. 5C

Accession No.	Sequence name
AHA46303	*Insectomime virus*
AHC55114	*Tunisvirus fontaine 2*
YP_009238536	*Brazilian marseillevirus*
AEA06871	*Lausannevirus*
ALH06713	*Port-Miou virus*
YP_009255194	*Tokyovirus A1*
YP_003406803	*Marseillevirus*
AGV01404	*Cannes 8 virus*
YP_009094549	*Melbournevirus*
AAQ09585	APMV

Table S4 Number of amoeba cells including virion factories (VF) of various types (PI 0–10 h p.i.) for Fig. S3

p.i. (h)	No VF	1 VF	2 or more VFs	Large VF	Total
0	300	0	0	0	300
2	281	14	5	0	300
4	178	98	24	0	300
6	176	112	11	1	300
8	82	176	25	17	300
10	20	172	37	71	300

Table S5 Number of amoeba cells including virion factories (VF) of various types (12–24 h p.i.) for Fig. S3

p.i. (h)	No VF	1 VF	2 or more VFs	Large VF	Total
0	400	0	0	0	400
12	32	130	13	225	400
18	35	88	30	247	400
24	30	26	16	328	400

Supplementary figure legends

Fig. S1. Various putative entry mechanisms of *Tokyovirus* into *Acanthamoeba* cells. *Tokyovirus* shows several morphological features in infected *Acanthamoeba* cells (5). Dotted circles indicate giant vesicles including several (or many) viral particles surrounding by membranes. Arrows indicate single particles in *Acanthamoeba* cells without membranes. Dotted arrows indicate single particles surrounded by membranes. Scale bar, 500 nm.

Fig. S2. *Tokyovirus* particles in the virion factory. Arrows indicate viral particles proceeding simultaneous assembly of DNA and capsid. Scale bar, 500 nm.

Fig. S3. Time course analysis of virion factories. *Tokyovirus* was used in PYG medium. The MOI was not determined, but was sufficient to cause a cytopathic effect (CPE) in all amoeba cells. Amoeba cells cultured in a 12-well microplate infected by *Tokyovirus* were harvested 2, 4, 6, 8, 10, and 12 h p.i., and were centrifuged at 150 × *g* for 5 min at room temperature, following by washing twice with PBS. After centrifugation as above, cells were suspended in 200 μl of methanol in 1.5 ml microtubes, and were incubated for 10 min at room temperature. One drop of cell suspension was dripped onto a glass slide and completely air-dried. Dried cells were incubated with 500 ng/ml of DAPI for 50 s and were then immediately washed twice with PBS. Stained cells were mounted in Vectashield (Vector Laboratories Inc.), and visualized using a fluorescence microscope (BX50; Olympus Corp.). In another experiment, *Tokyovirus*-infected amoeba cells were also stained with DAPI as described above at 12, 18, and 24 h p.i. Amoeba cells containing virion factories were manually counted up to 300 or 400 cells. The ratio of amoeba cells containing each type of virion factory (Large VF, 2 or more VFs, 1 VF, and no VF) were tested at 0, 2, 4, 6, 8, 10, 12, 18, and 24 h p.i. Experiments at 2 h to 10 h p.i. and those at 12 h to 24 h p.i. were performed separately. Inset fluorescent images show typical amoeba cells possessing virion factories of different types.

Fig. S4. Genome comparison between different subclade members of *Marseilleviridae* using dot plot analysis. (A) Dot plot analysis between *Melbournevirus* (subclade A) and *Brazilian marseillevirus* (subclade D). (B) Dot plot analysis between *Tunisvirus* (subclade C) and *Brazilian marseillevirus* (subclade D). (C) Dot plot analysis between *Tunisvirus* (subclade C) and *Lausannevirus* (subclade B).

Fig. S5. Genome comparison between the same subclade members of *Marseilleviridae* using dot plot analysis. (A) Dot plot analysis between *Marseillevirus* and *Melbournevirus* (both subclade A). (B) Dot plot analysis between *Marseillevirus* and *Cannes 8 virus* (both subclade A). (C) Dot plot analysis between *Melbournevirus* and *Cannes 8 virus* (both subclade A). (D) Dot plot analysis between *Lausannevirus* and *Port-miou virus* (both subclade B). (E) Dot plot analysis between *Tunisvirus* and *Insectomime virus* (both subclade C).

Fig. S6. Unrooted maximum-likelihood phylogenetic tree of 30 DNA polymerase sequences constructed using MEGA6 software (26). The tree was reconstructed based on an alignment (634 sites) derived from the full-length alignment, in which any

column containing a gap had been discarded. Numbers at the branch points denote percent bootstrap values. Accession numbers of respective sequences are listed in Table S1. Alphabets A, B, and C indicate subclades of *Marseilleviridae*, respectively.

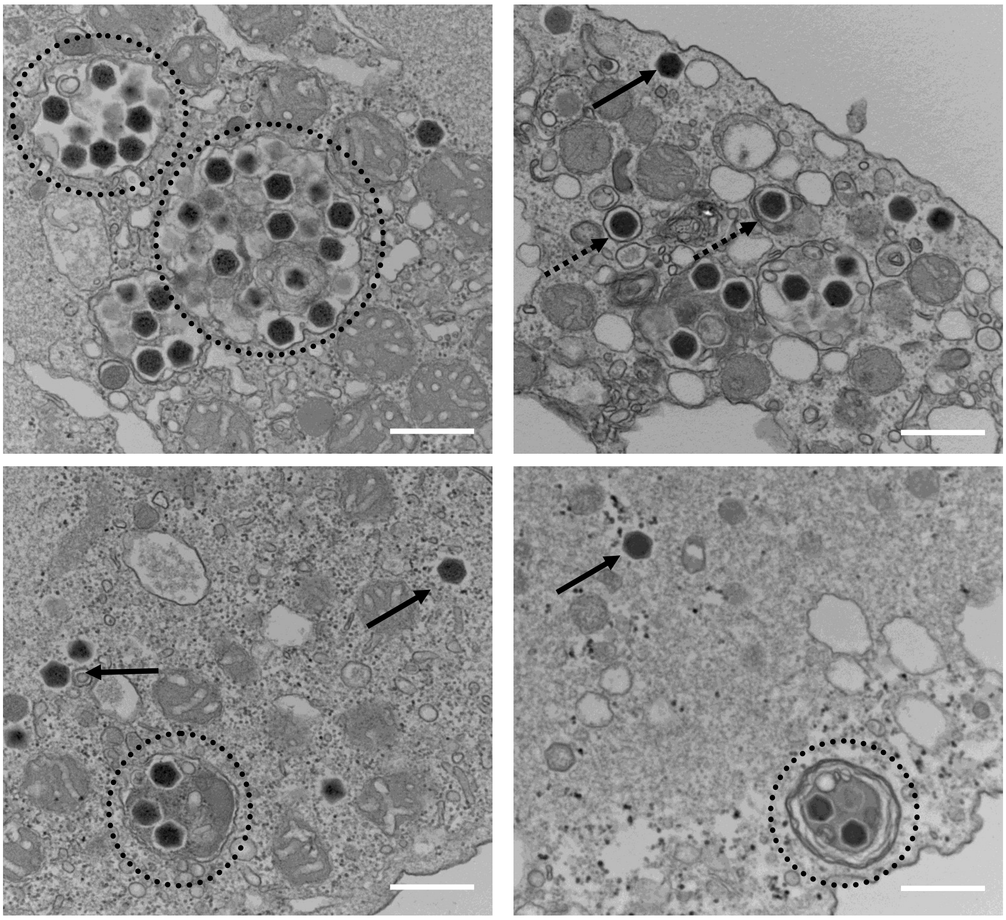

Fig. S1
Masaharu Takemura

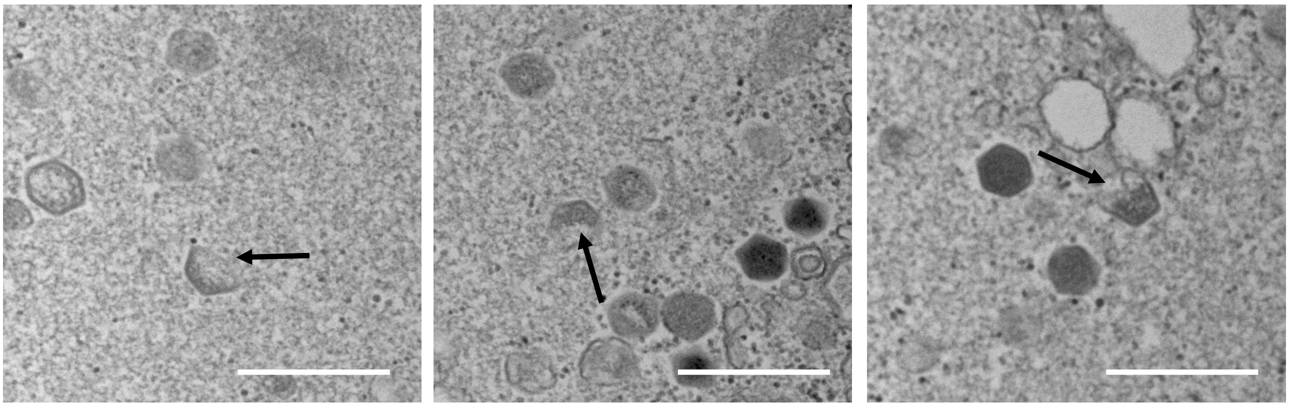

Fig. S2
Masaharu Takemura

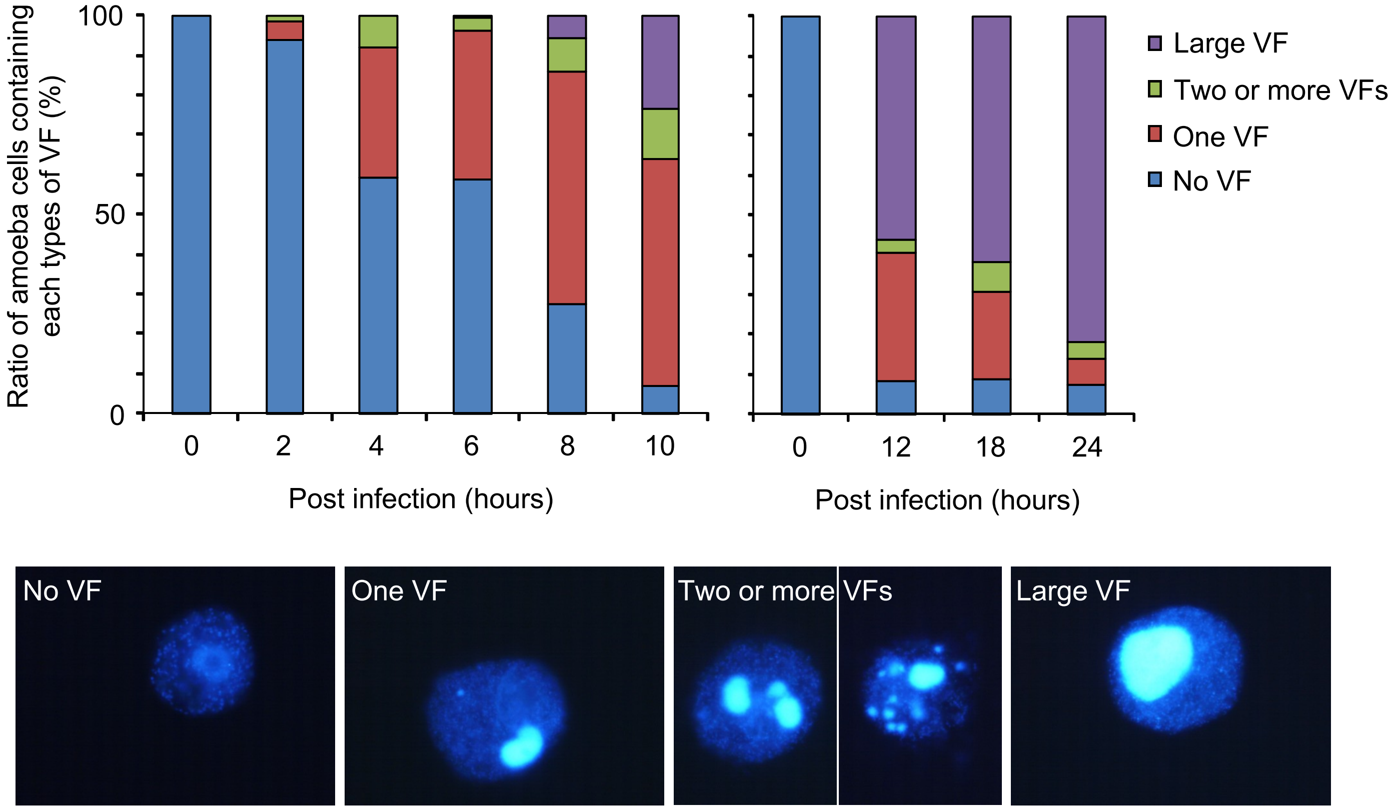

Fig. S3
Masaharu Takemura

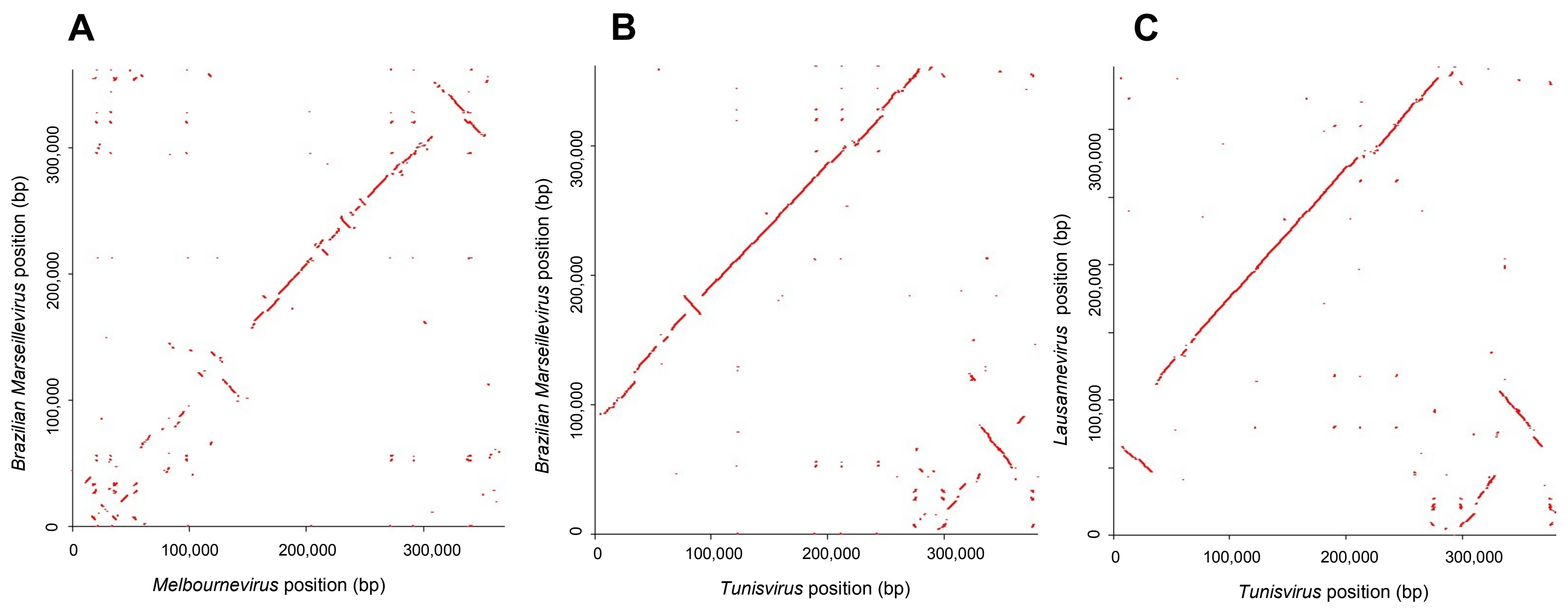

Fig. S4

Masaharu Takemura

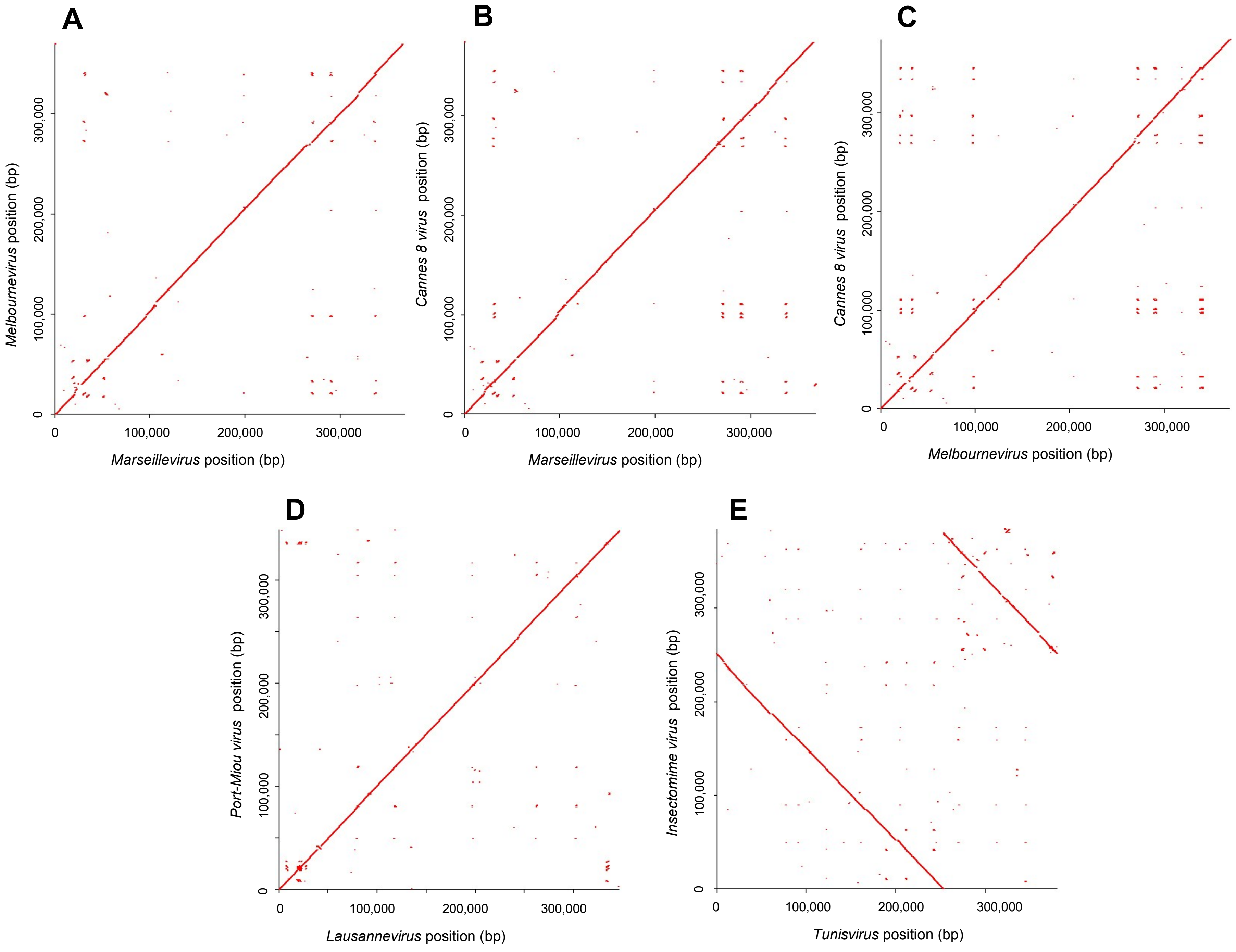

Fig. S5

Masaharu Takemura

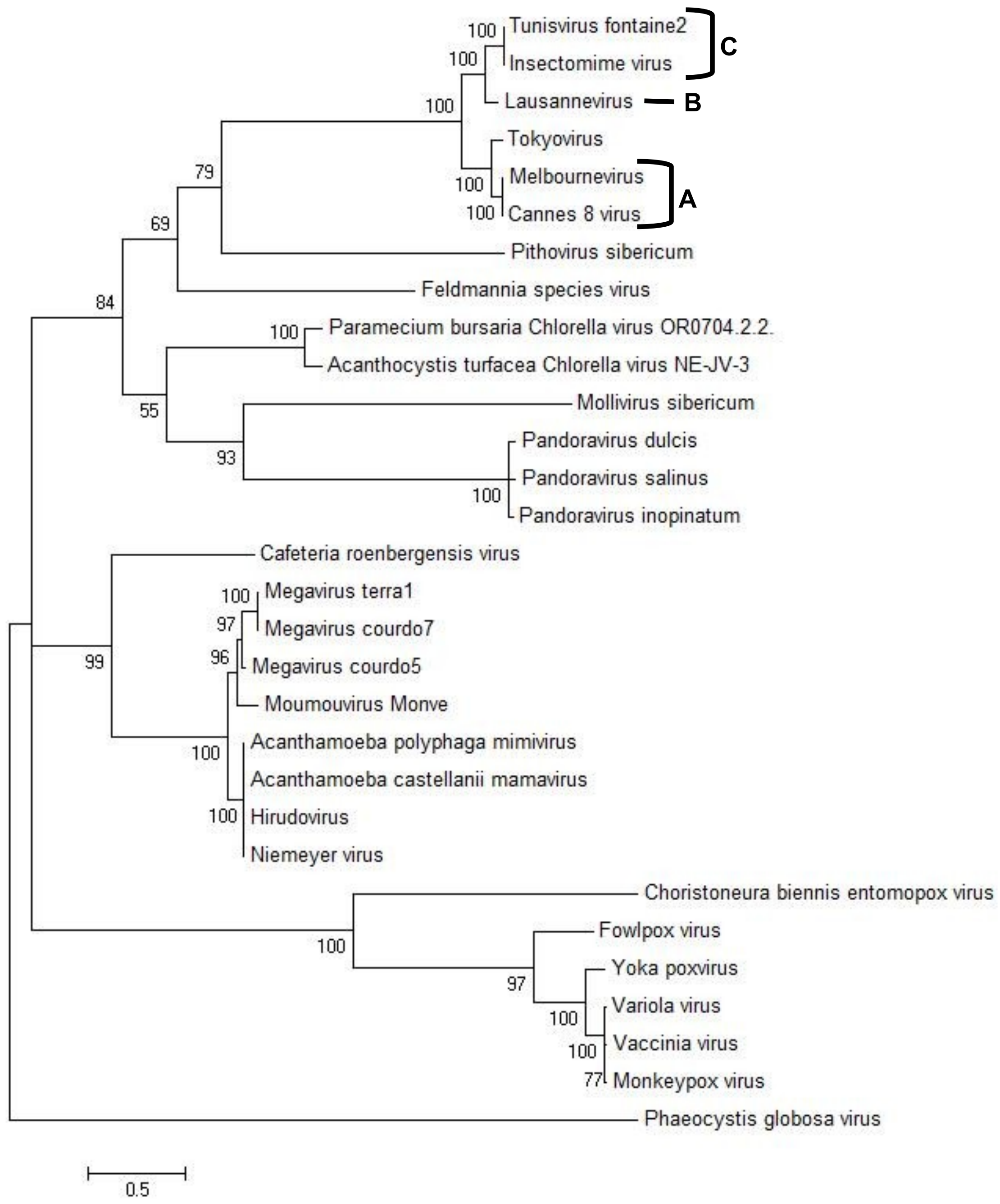

Fig. S6
Masaharu Takemura
